# Distinct Hepatic Metabolic Reprogramming in Acute and Chronic Sleep Deprivation and the Protective Effects of the Chalcone Analogue TAK

**DOI:** 10.3390/ijms26083485

**Published:** 2025-04-08

**Authors:** Yifang Wang, Yachong Hu, Pengxiao Wang, Ranrui Hu, Zhongqi Chen, Tiantian Zhang, Jiankang Liu, Mami Noda, Jiangang Long, Yunhua Peng

**Affiliations:** Center for Mitochondrial Biology and Medicine, The Key Laboratory of Biomedical Information Engineering of Ministry of Education, School of Life Science and Technology, Xi’an Jiaotong University, Xi’an 710049, China; yifang@stu.xjtu.edu.cn (Y.W.); huyachong@126.com (Y.H.); 15729012288@139.com (P.W.); hrruse@163.com (R.H.); zhongqic@med.umich.edu (Z.C.); sanjie1993@126.com (T.Z.); j.liu@mail.xjtu.edu.cn (J.L.); maminoda39@gmail.com (M.N.)

**Keywords:** acute sleep deprivation, chronic sleep deprivation, liver, TAK

## Abstract

The prevalence of sleep deprivation is increasing worldwide. Despite the vital roles that the liver plays in metabolism and immune response, hepatic dysfunctions in acute sleep deprivation (ASD) and chronic sleep deprivation (CSD) remain underexplored. Additionally, the effects of the newly developed chalcone analog, 1-(2,3,4-trimethoxyphenyl)-2-(3,4,5-trimethoxyphenyl)-acrylketone (TAK), were evaluated as a potential therapeutic chemical for mitigating SD-induced hepatic damage. A modified multi-platform method was employed to prepare animal models of 72 h ASD and 21-day CSD in rats. TAK (50 mg/kg/day) was administered through irrigation starting one week before the experiment and continuing until the end. ASD triggered hepatic lipid accumulation and inflammation, whereas CSD resulted in pathological portal area expansion and fibrosis, with comparatively fewer disturbances in liver metabolism and inflammation. TAK effectively alleviated ASD-induced disruptions in glycogen synthesis via PI3K/AKT/GSK3/GYS2 pathways, abnormal lipid accumulation via SREBP1/FASN/ACC, liver inflammation by balancing M1 and M2 macrophages, and liver fibrosis induced by ASD/CSD. This study provides valuable insights into the different mechanisms of liver damage induced by severe ASD and mild CSD. Additionally, TAK has been proposed as a potential therapeutic strategy for ultimate SD-related hepatic complications.

## 1. Introduction

Sleep is one of the most essential physiological processes for humans. Some special occupational groups often experience short-term continuous sleep deprivation (acute sleep deprivation, ASD), while others encounter long-term intermittent sleep deprivation (chronic sleep deprivation, CSD) in their daily lives. According to the Chinese Sleep Research Report, the average sleep duration for Chinese people is approximately 7 ± 1.35 h per night in 2023 [1]. Nearly 35% of adults sleep less than 7 h per day, and approximately 60 million US adults suffer from sleep disorders [2], which can predispose individuals to central and peripheral diseases, including hepatic damage [3], insulin resistance [4], atherosclerosis [5], memory impairment [6], inflammatory disorders [7], and oxidative damages [8].

As a viral hub for numerous physiological processes, including immune function, macronutrient metabolism, glucose and lipid homeostasis, and drug metabolism, the liver is particularly vulnerable to dysfunction under stress conditions [9]. Sleep disorders are associated with an increased risk of liver cancer and mortality [10]. The 6 h of sleep deprivation were affiliated with the development of glucose intolerance, enhanced hepatic glucose production, and hepatic steatosis [4]. By implementing a schedule of 10 days with 20 h of wakefulness followed by 4 h of rest, liver metabolism of glutathione, fructose, mannose, and pyruvate was impaired [11]. A few findings on immune system changes after SD are debatable due to the varying models and sampling times of SD applied. One night of SD significantly altered circulating immature neutrophils in healthy men [12], and 72 h SD reduced the content of cytotoxic T cells (CD8+) counts in mouse spleen [13]. CSD mainly affects immunity in the blood and spleen by increasing the levels of CD3+ T lympgocytes cells, CD4+ T helper cells, and natural killer cells and decreasing the levels of CD8+ in the mice [14]. CSD also decreased the number of cytotoxic cells in mice, impaired the antitumor immune response, and accelerated the rate of lung metastasis [15]. The differential injury triggered by ASD and CSD also remains largely unknown. There is a significant gap in our understanding of how ASD and CSD specifically impact the metabolic and immune systems, particularly regarding their underlying mechanisms and potential therapeutic interventions for liver damage.

Previous studies have indicated that chalcone derivatives with electron-donating groups, such as hydroxyl, methoxy, and methyl substitutions on the benzene ring, may exhibit stronger biological activity [16]. The 1-(2,3,4-trimethoxyphenyl)-2-(3,4,5-trimethoxyphenyl)-acrylketone (TAK) has shown therapeutic potential in mitigating oxidative stress and neuronal damage induced by scopolamine, while overcoming low bioavailability in its natural form [17]. In this study, we aim to investigate whether TAK could also protect livers from tissue damage induced by SD and understand the therapeutic potential of TAK.

We employed the Multi-Modal Protocol Model (MMPM) to establish a 72 h ASD model and a 21-day CSD model. We assess the physiological and pathological alterations, encompassing modifications in glucose and lipid metabolism, immune function, and histological changes in the liver. We aim to elucidate the differential liver damage triggered by ASD and CSD and investigate the hepatic protective potential of TAK.

## 2. Results

### 2.1. Body Weight Changes, Hormonal Disorders, and Rhythm Disturbances in SD and the Effects of TAK

**Figure 1:**
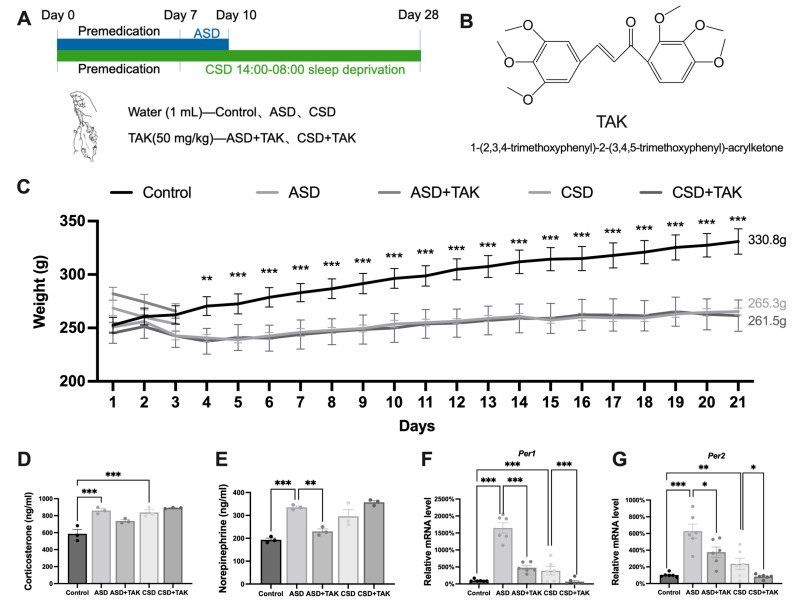
Sleep deprivation-induced rhythm perturbations and TAK effectively reverse it. (**A**) The schematic procedure of drug treatments and experiment design of ASD and CSD. (**B**) Chemical structure of TAK. (**C**) Body weight (n = 6). (**D**) Serum norepinephrine (n = 3). (**E**) Serum corticosterone (n = 3). (**F**) Liver *Per1* expression (n = 6). (**G**) Liver *Per2* expression (n = 6). Data are presented as means ± SEM. Statistical analysis was performed using one-way ANOVA with Dunnett’s test, performed with GraphPad Prism 10. Significance levels are indicated as follows: * *p* < 0.05, ** *p* < 0.01, *** *p* < 0.001.

We utilized MMPM (modified multi-platform method), an experimental setup with a small platform within a water environment, to subject rats to the SD model and investigate the hepatoprotective effects of TAK. The experimental protocol is shown in Figure 1A. The chemical structure of 1-(2,3,4-trimethoxyphenyl)-2-(3,4,5-trimethoxyphenyl)-acrylketone (TAK) was shown in Figure 1B. Compared to the control group, there was no significant decrease in body weight among the ASD group and the TAK-treated group, suggesting that ASD and TAK do not affect rat body weight. The rat’s body weight declined significantly after the 4th day of CSD, which was not mitigated by TAK treatment (Figure 1C). Subsequently, we measured indicators related to circadian rhythm and stress-related hormones. Both ASD and CSD caused an increase in the levels of corticosterone (Figure 1D) and norepinephrine (Figure 1E). TAK treatment did not suppress the corticosterone level in both ASD and CSD (Figure 1D) but significantly restored the levels of norepinephrine only after ASD (Figure 1E). In terms of circadian rhythm, elevated mRNA levels of the rhythm-regulating gene period circadian regulator 1 (*Per1*) and *Per2* were observed, which were significantly downregulated by TAK after ASD and CSD (Figure 1F,G). In the case of ASD, despite minimal effects on body weight, pronounced disruptions in hormone levels (Figure 1D,E) and circadian rhythm were supposed to be disturbed (Figure 1F,G). In contrast, body weight was 72 g less than control rats on the last day of CSD (Figure 1C), and less rhythmicity changed. TAK showed no influence on the body weight either in ASD or in CSD and served as a beneficial modulator for increased norepinephrine in ASD and increased expression of *Per1* and *Per2* mRNAs in both ASD and CSD.

### 2.2. Glycogen Synthesis Was Inhibited Following ASD and Was Rescued by TAK Possibly via the PI3K/AKT/GSK3/GYS2 Pathway

**Figure 2:**
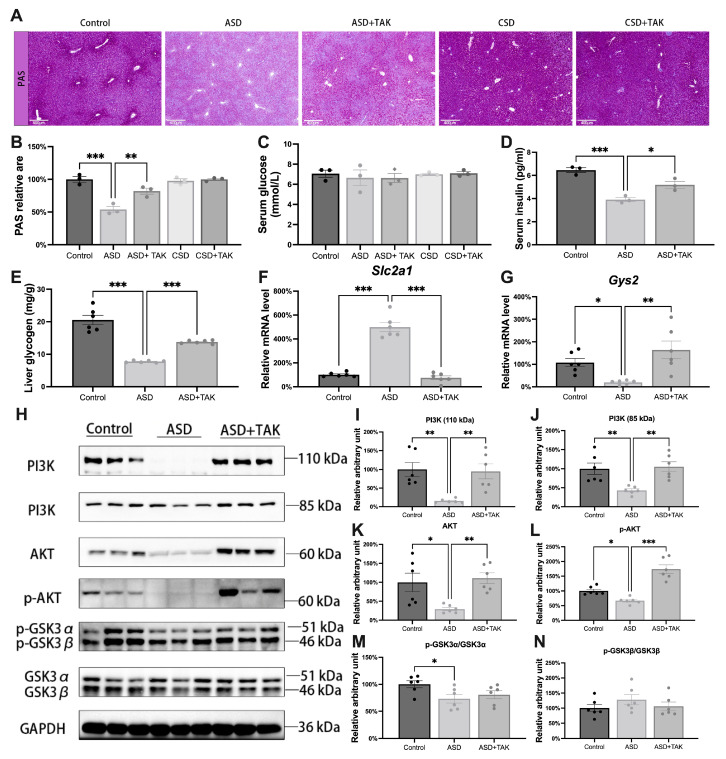
ASD inhibited glycogen synthesis, and TAK resumed via the PI3K/AKT/GSK3/GYS2 pathway. (**A**) PAS staining (Scale bar: 400 μm, n = 3). (**B**) Quantification of PAS staining (n = 3). (**C**) Serum glucose levels (n = 3). (**D**) Serum insulin content (n = 3). (**E**) Liver glycogen content (n = 6). (**F**) Liver *Slc2a1* expression (n = 6). (**G**) Liver *Gys2* expression (n = 6). (**H**) Western blotting image. (**I**–**N**) Quantification of relative protein content of PI3K (110 kDa), PI3K (85 kDa), p-AKT, AKT, p-GSK3α/GSK3α, p-GSK3β/GSK3β in rat liver (n = 6). Data are presented as mean ± SEM. Statistical analysis was performed using one-way ANOVA with Dunnett’s test, performed with GraphPad Prism 10. Significance levels are indicated as follows: * *p* < 0.05, ** *p* < 0.01, *** *p* < 0.001.

Glycogen is an important energy source, and its synthesis and breakdown affect vital activities. After ASD, we found a significant decrease in glycogen (Figure 2A,B), while serum glucose levels were not affected after ASD and CSD (Figure 2C). However, serum insulin content in serum (Figure 2D) and liver glycogen (Figure 2E) were significantly impaired after ASD, and both were restored by application of TAK. ASD significantly upregulated the mRNA expression of glucose transporter 1 (solute carrier family 2 member 1, *Slc2a1*) (Figure 2F). After the application of TAK, ASD-induced upregulation of *Slc2a1* was completely attenuated to the control level (Figure 2F). On the other hand, mRNA expression level of glycogen synthase 2 (*Gys2*) was significantly decreased after ASD, which was completely recovered by TAK (Figure 2G). To detect the effects of ASD and TAK on the downstream glucose metabolic pathways, the expression levels of proteins in the phosphoinositide 3-kinase (PI3K)/protein B (AKT)/glycogen synthase kinase-3 (GSK3) pathway regulating glycogen synthesis were analyzed by Western blot (Figure 2H). AKT is a crucial regulatory factor for cell survival and metabolism. When activated by signals such as insulin, AKT phosphorylates glycogen synthase kinase 3 (GSK3), rendering it inactive. Inactive GSK3 no longer phosphorylates glycogen synthase, thereby promoting glycogen synthesis. GSK3, a critical downstream element of the PI3K/AKT cell survival pathway, was initially identified as an enzyme that regulates glycogen synthesis in response to insulin [18] and is a ubiquitously expressed serine/threonine protein kinase that phosphorylates and inactivates glycogen synthase. We found that ASD caused a significant downregulation of PI3K, AKT, and p-AKT protein expression levels (Figure 2I–L), leading to the downregulation of p-GSK3α/GSK3α (Figure 2M), but not affecting p-GSK3β/GSK3β (Figure 2N). Inactivation of phosphorylated GSK3α and dephosphorylated GSK3α phosphates GYS2, causing its inactivation and thereby reducing glycogen synthesis. After TAK treatment, PI3K, AKT, p-AKT, and p-GSK3α/GSK3α were significantly restored (Figure 2I–N), suggesting that TAK promotes glycogen synthesis by elevating GYS2 expression levels. In summary, ASD increased liver glucose absorption capacity while concurrently reducing glycogen synthesis, indicating glucose entered other metabolic pathways rather than glycogen synthesis.

### 2.3. Glucose Converted to Lipid Accumulation After ASD: Hindered by TAK Treatment

**Figure 3:**
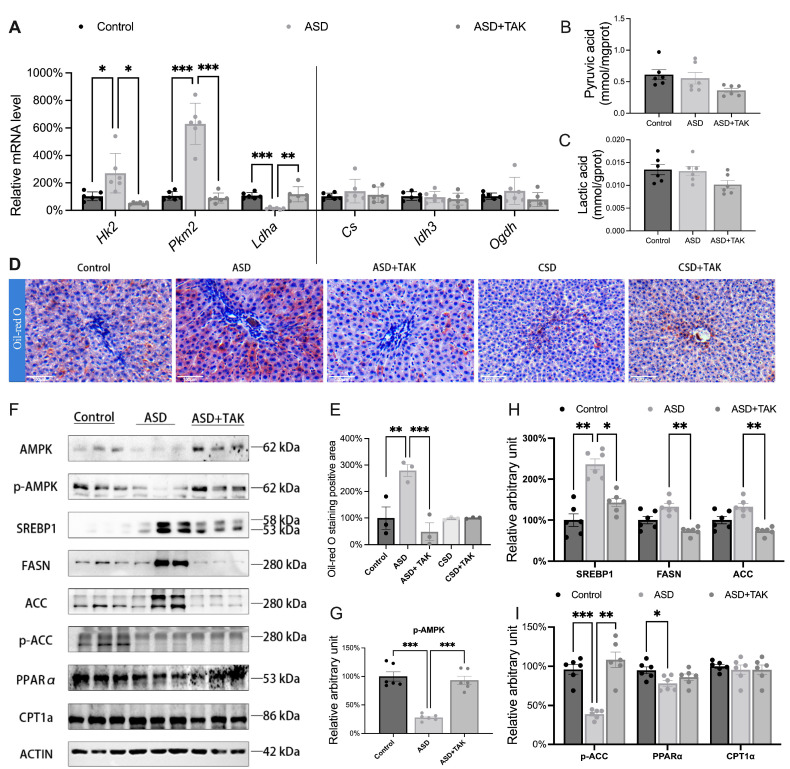
Glycose transformed into lipid through accelerated glycolysis after ASD and TAK alleviated lipid accumulation. (**A**) Relative gene expression code key enzymes of glycosis and TCA cycle, *Hk2*, *Pkm2*, *Ldha*, *Cs*, *Idh3*, and *Ogdh* (n = 6). (**B**) Liver pyruvate content (n = 6). (**C**) Liver lactic acid content (n = 6). (**D**) Oil-red O staining (Scale bar: 100 μm, n = 3). (**E**) Quantifications of Oil-Red O staining (n = 3). (**F**) Western blotting image and (**G**–**I**) statistics analysis on relative protein content of p-AMPK, SREBP1, FASN, ACC, p-ACC, PPARα, and CPT1α (n = 6). Data are presented as mean ± SEM. Statistical analysis was performed using one-way ANOVA with Dunnett’s test, performed with GraphPad Prism 10. Significance levels are indicated as follows: * *p* < 0.05, ** *p* < 0.01, *** *p* < 0.001.

Glycogen synthesis was inhibited after ASD, and glucose was converted to lipid synthesis through glycolysis and tricarboxylic acid (TCA). We first tested indicators of glycolysis and the TCA cycle. For the two rate-limiting enzymes of glycolysis, we found that ASD significantly upregulated hexokinase 2 (*Hk2*) and pyruvate kinase M2 (*Pkm2*) (Figure 3A), yet pyruvate content remained unchanged (Figure 3B), suggesting that glucose entered glycolysis without causing pyruvate accumulation. Additionally, we measured the levels of lactate dehydrogenase A (*Ldha*) and lactate, finding that ASD significantly down-regulated *Ldha* (Figure 3A), while there was no significant change in lactate content (Figure 3C), indicating that pyruvate continued to enter the TCA cycle. After TAK treatment, *Hk2*, *Pkm2*, and *Ldha* were all significantly restored (Figure 3A). We measured the mRNA levels of three rate-limiting enzymes in the TCA cycle, such as citrate synthase (*Cs*), isocitrate dehydrogenase (*Idh3*), and α-ketoglutarate dehydrogenase (*Ogdh*), and there were no significant differences among them (Figure 3A).

Using Oli-red O, a lysochrome (fat-soluble dye) diazo dye for staining of neutral triglycerides and lipids (Figure 3D), it was observed that lipids accumulated only after ASD, but not CSD (Figure 3E). We then tested proteins related to lipid synthesis and oxidation (Figure 3F). AMPK is a cellular energy sensor that becomes activated in response to low energy levels within the cell. Once activated, AMPK can inhibit the nuclear maturation of steroid regulatory element-binding protein 1 (SREBP1) through phosphorylation. This process reduces the transcriptional activation of genes related to lipid synthesis, thereby suppressing lipid production. Under the influence of ASD, AMPK and p-AMPK were significantly downregulated (Figure 3F–H). However, it caused an upregulation of transcription factors regulating lipid synthesis, sterol regulatory element-binding protein 1 (SREBP1), which in turn led to a significant upregulation of the enzymes catalyzing lipid synthesis, fatty acid synthase (FASN), and acetyl-CoA carboxylase (ACC) (Figure 3F,H). ACC, a substrate of AMPK, underwent phosphorylation modifications that inhibited lipid synthesis, and ASD led to the downregulation of p-ACC (Figure 3F,I). Also, peroxisome-activated receptor α (PPARα), a nuclear receptor-type transcription factor regulating fatty acid oxidation and inflammation in the liver, was significantly downregulated by ASD (Figure 3F,I). On the other hand, ASD had no significant effect on the expression of liver-type carnitine palmitoyl transferase 1A (CPTIA), indicating that PPARα regulated fatty acid oxidation through other enzymes. (Figure 3F,I). In summary, glucose is converted into lipid and subsequently stored in the liver after ASD; however, TAK alleviates hepatic lipid accumulation by activating AMPK.

### 2.4. ASD-Induced Inflammation and Possible Anti-Inflammatory Effects of TAK

**Figure 4:**
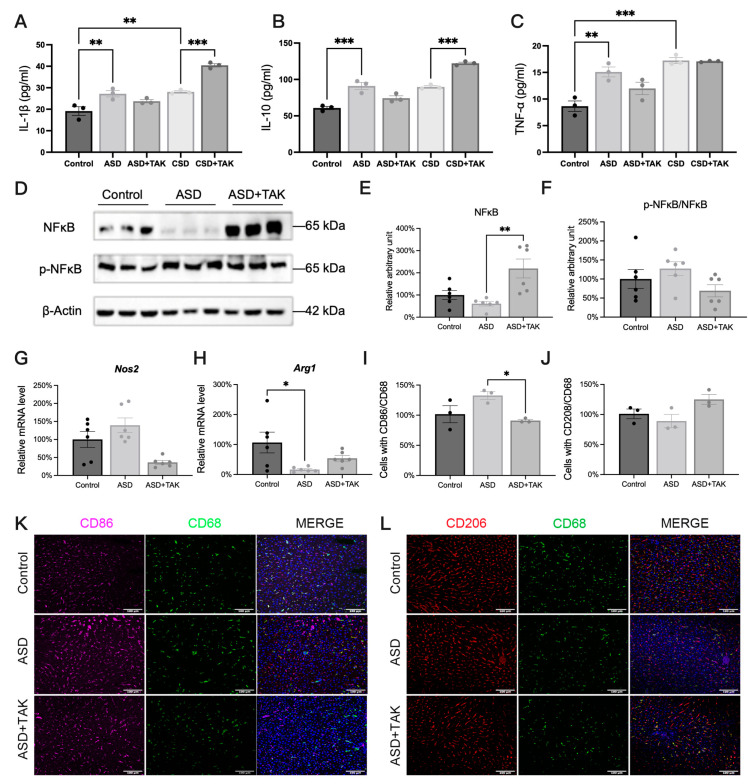
ASD activated inflammatory cytokine factor and improved after TAK. (**A**) Serum IL1 content (n = 3). (**B**) Serum IL10 content (n = 3). (**C**) Serum TNF-α content (n = 3). (**D**) Western blotting image and (**E**,**F**) statistics analysis on the relative protein content of NFκB and p-NFκB (n = 6). (**G**) Liver *Nos2* expression (n = 6). (**H**) Liver *Arg1* expression (n = 6). (**I**) Statistics of CD86/CD68 staining (n = 3). (**J**) Statistics of CD206/CD68 staining (n = 3). (**K**) Immunofluorescence of CD86 and CD68 (Scale bar: 100 μm, n = 3). (**L**) Immunofluorescence staining for CD206 and CD68 (Scale bar: 100 μm, n = 3). Data are presented as mean ± SEM. Statistical analysis was performed using one-way ANOVA with Dunnett’s test, performed with GraphPad Prism 10. Significance levels are indicated as follows: * *p* < 0.05, ** *p* < 0.01, *** *p* < 0.001.

In terms of inflammation, we examined cytokine levels in the serum. ASD increased the pro-inflammatory factors IL-1β and TNF-α (Figure 4A,C), while CSD increased IL-1β and TNF-α significantly (Figure 4A,C), but not IL-10 (Figure 4B). TAK treatment did not significantly affect the ASD-increased cytokine levels. In CSD, TAK increased the level of IL-1β and IL-10 (Figure 4A,B). To further investigate the cytokine production, we measured the expression level of NFκΒ and p-NFκΒ. The expression of NFκΒ looked downregulated in ASD (Figure 4D), though not statistically different (Figure 4E), while TAK treatment significantly increased the NFκΒ (Figure 4D,E). Nevertheless, the ratio, p-NFκΒ/NFκΒ, was not affected by either ASD nor ASD with TAK (Figure 4F). We also checked the mRNA levels of inflammation-related factors such as inducible nitric oxide synthase (*Nos2*), an M1 macrophage maker (Figure 4G), arginase 1 (*Arg1*), and an M2 macrophage maker (Figure 4H). Arg1 was significantly decreased by ASD, which was not affected by TAK (Figure 4H). To distinguish macrophages, we used CD68, CD86, and CD206 as the marker representative of total macrophages, M1 macrophages, and M2 macrophages, respectively. Through co-immunofluorescence staining of CD86^+^/CD68^+^ (indicating M1 macrophages, Figure 4K) and CD206^+^/CD68^+^ (indicating M2 macrophages, Figure 4L), there was a tendency for more M1 macrophages (Figure 4I) and fewer M2 macrophages after ASD (Figure 4J), which was not significant changes, as observed in Figure 4H. TAK significantly inhibited the number of M1 macrophages (Figure 4I), suggesting that TAK may exert anti-inflammation through regulating the M1/M2 macrophage ratio.

### 2.5. SD Stimulated Hepatic Portal Area Expansion and Fibrosis, Which Were Rescued by TAK

**Figure 5:**
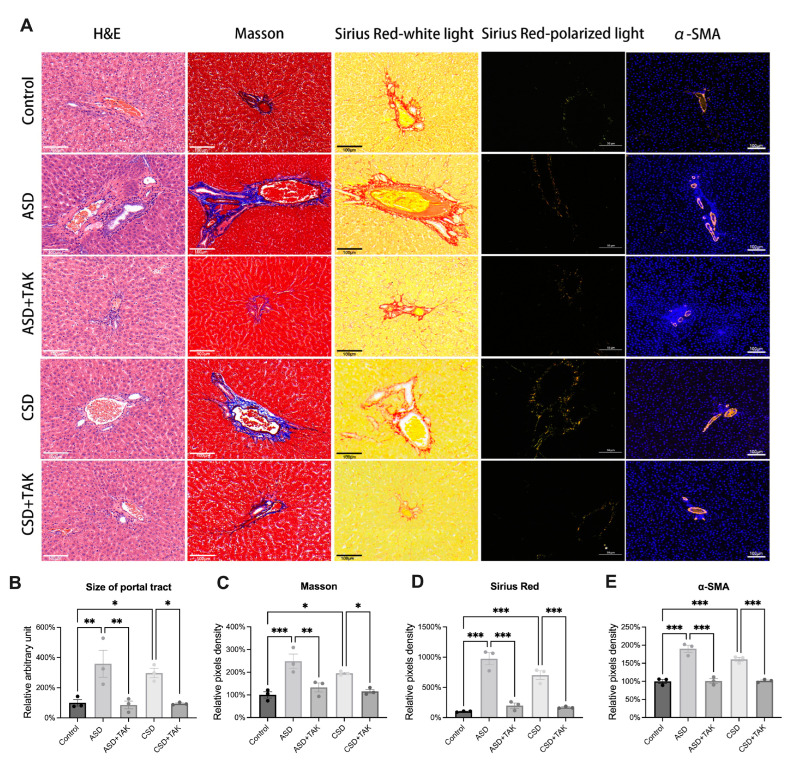
The liver portal area fibrosis following ASD and CSD was alleviated by TAK. H&E (Scale bar: 100 μm, n = 3), Masson (Scale bar: 100 μm, n = 3), Sirius red-white light (Scale bar: 100 μm, n = 3), Sirius red-polarized light (Scale bar: 50 μm, n = 3) and α-SMA staining (Scale bar: 100 μm, n = 3) of liver tissue (**A**). Quantification of size of portal tract, Masson staining, Sirius red staining and α-SMA staining (n = 3) (**B**–**E**). Data are presented as mean ± SEM. Statistical analysis was performed using one-way ANOVA with Dunnett’s test, performed with GraphPad Prism 10. Significance levels are indicated as follows: * *p* < 0.05, ** *p* < 0.01, *** *p* < 0.001.

From the H&E staining results, we observed lymphocytes in the portal tract spill out into the limiting plate of surrounding hepatocytes only after ASD (Figure 5A,B). Given the observed glucose and lipid metabolism disorder, along with the inflammations associated with ASD, we wondered if there was any other severe damage to the liver. We applied Masson (Figure 5A,C), Sirius red (Figure 5A,D), and α-smooth muscle actin (α-SMA) staining (Figure 5A,E) to determine the hepatic fibrosis levels. The results indicated an expansion of the portal area and the induction of portal area fibrosis by both ASD and CSD (Figure 5B).

## 3. Discussion

SD is a multifaceted pathological process that often leads to multiple organ injuries, posing a global health threat. To estimate the level of threat, most rats die after 5 weeks SD, which only 7% to 10% of rats could survive [19]. In this study, we revealed the distinct liver damage patterns induced by ASD and CSD and demonstrated the protective effects of TAK, a chalcone analog, against SD-induced hepatic damage.

Rats lost 70 g of weight at the end of CSD, while less weight loss happened to ASD. It is reported that stress-induced cortisol and norepinephrine elevations promote lipid synthesis and reduce insulin [20,21]. It is similar to previous studies in that circadian rhythm, cortisol, and norepinephrine release were significantly increased after ASD and less increased after CSD, which insinuates drastic changes happened after ASD.

Previous studies have reported an increased lipid accumulation in the liver following SD [4], yet its underlying mechanisms remained elusive. From the results of Oil-red O staining, PAS staining, and H&E staining, there were no significant changes observed in glucose and lipid metabolism or inflammatory disorders in the livers of rats following chronic sleep deprivation. Given the more pronounced changes in staining results after ASD, we primarily focus on the mechanisms of TAK protective effect in the livers of rats subjected to acute sleep deprivation.

Notably, we found that more glucose uptake happened in hepatocytes via increased expression of *Slc2a1*, but less glycogen was synthesized and accumulated in the liver. Glucose is preferentially stored in the form of glycogen rather than fat under health conditions [22], unlike our observations in the ASD model. In diabetes, neurodegenerative diseases and other conditions and the dysregulation of the AKT/GSK3 signaling pathway may result in impaired glycogen synthesis and abnormal glucose regulation.

The abnormal ratio of AMPK to SREBP1 is closely associated with the onset and progression of obesity, fatty liver disease, atherosclerosis, and other metabolic disorders. Uridine diphosphate glucose (UDPG), an intermediate in glycogen synthesis, is transported to the Golgi apparatus and binds to S1P protease, inhibiting the cleavage of SREBP and lipid synthesis, promoting glycogen synthesis [22]. In our study, glucose transferred into the hepatocytes was not directed for glycogen synthesis, but for lipid synthesis. β-fatty acid oxidation was also suppressed by ASD, presumably contributing to the observed lipid accumulation.

Our results indicate that ASD induces an imbalance in glucose and lipid metabolism, which provides details into the mechanisms of ASD-induced lipid accumulation. The results of the hematoxylin and eosin (HE) staining indicate that the livers of rats subjected to acute sleep deprivation did not display characteristics typical of ballooning or fatty liver degeneration. While there were indications of inflammatory infiltration and fibrosis in the portal area, the HE staining results showed that the arrangement of liver cells was compact and orderly. However, these findings did not fulfill the criteria for fatty liver degeneration. Furthermore, the molecular signaling pathway associated with liver lipid accumulation following sleep deprivation was preliminarily analyzed based solely on the lipid component accumulation observed in the oil red staining results. It is important to note that oil red staining lacks sufficient sensitivity for cholesterol detection, as it primarily targets neutral fats and is not specific to cholesterol. Additionally, there was no significant change in total cholesterol (T-CHO) content in the rats’ livers (Supplementary Materials).

TAK alleviated metabolism disturbances after ASD by promoting glycogen synthesis through the PI3K/AKT/GSK3/GYS2 pathway and reducing lipid accumulation via the SREBP1/FASN/ACC. These findings not only provide a mechanistic understanding of TAK’s protective effects but also open up avenues for the development of targeted interventions aimed at mitigating the metabolic burden associated with SD.

Inflammation is closely related to abnormal lipid metabolism, and inflammatory mediators affect the homeostasis of lipid function from aspects such as lipid synthesis, secretion, and oxidation [23]. In terms of immune response in the liver, various cell types, such as Kupffer cells (KCs), hepatic stellate cells (HSCs), hepatic sinusoid endothelial cells, and dendritic cells, are involved, making the liver a crucial regulator of immune response in the human body [24]. Accumulating evidence suggests that SD could elevate inflammatory factors, reflecting disturbed physiological functions and disease progressions [25]. KCs are specialized macrophages located in the liver and are part of the mononuclear phagocytic system. Hepatic inflammation and fibrosis depend on the balance regulation between pro-inflammatory M1 and anti-inflammatory M2 macrophage subpopulations [26]. IL-1β and TNF-α are the key inflammatory cytokines that can amplify various biological responses, including apoptosis, autophagy, and organ injury responses [27]. Our data showed significant elevations of serum IL-1β and TNF-α post-ASD, along with increased p-NFκB, imbalanced M1/M2 macrophage ratios, and created lymphocyte spillage from portal tracts into surrounding hepatocytes.

The severe physiological changes faded with prolonged sleep deprivation time because of the powerful self-regulation ability. Liver cells (mainly liver parenchymal cells) in the resting phase will re-enter the cell cycle for proliferation even after undergoing 2/3 liver resection or acute toxicity injury, generating new liver cells and restoring full liver quality and function in less than two weeks. It is worth noting that the liver fibrosis induced by SD persisted, even though we only observed the portal area fibrosis. Sleep disorders have been implicated in the progression of chronic liver diseases, particularly non-alcoholic fatty liver disease and alcoholic fatty liver disease [3]. Given the liver’s remarkable regenerative capabilities, early detection of hepatic damage is very difficult, resulting in clinically significant liver dysfunction that poses irreversible damage. It is valuable to find that liver pathology changes happen after SD, to explain why sleep disorder affects the process of liver disease.

As previous research reported, metabolism disorders [28] and inflammations [29] could exacerbate hepatic fibrosis. Significant changes in liver vascular structures occur in patients with liver fibrosis and cirrhosis, particularly in HSCs and KCs, indicating their close associations with the progression of liver fibrosis [30,31]. We have observed that ASD and CSD could induce hepatic portal area fibrosis. HSCs are stimulated to differentiate into proliferative and fibrotic myofibroblasts in response to liver damage, by which they acquire a series of common features that mitigate injuries and initiate the fibrosis process [32]. Activation in primary culture enhances glucose transportation and glycolytic activities. GLUT1, GLUT2, and GLUT4 transporters are expressed in both activated and immortalized rat HSCs, and their expression levels can be modulated by high extracellular glucose concentrations or purinergic signals [33]. After HSCs are activated, HSCs upregulate glycolysis to fulfill the energy demands of differentiating into the myofibroblast phenotype. Such reports may explain why ASD promoted hepatocytes to glucose uptake and accelerated glycolysis.

TAK is a flavonoid compound characterized by a three-carbon atom structure, which closely resembles that of anthocyanins; it has significant medicinal potential for alleviating the complex physiological changes induced by sleep disorders. Our investigation revealed that TAK attenuated inflammation reactions, including alleviating serum IL-1 and TNF-α production after ASD, inhibiting NFκB activities, and restoring M1/M2 macrophage balance post-ASD. The administration of TAK triggered a complex immune response following chronic sleep deprivation. Further experimental verification is necessary to investigate the role and mechanisms of TAK in the chronic inflammation associated with sleep deprivation.

This research significantly advances our comprehension of TAK’s pivotal protective function in mitigating metabolic disorders within liver tissue, specifically in restoring the delicate balance between glucose and lipid metabolism. The molecular mechanism underlying this physiological phenomenon deserves further exploration, as this may expand the applications of TAK on other pathological processes while providing novel drug targets to mitigate SD-induced pathological changes. While the therapeutic potential of TAK in addressing metabolic disorders and liver damage associated with ASD is promising, its effects on other metabolic aspects require further validation.

## 4. Materials and Methods

### 4.1. Animals

All rats, male, about 180 g, and 8 weeks old, were purchased from the Institutional Animal Care and Use Committee of Xi’an Jiaotong University. Rats were maintained in a specific room with constant humidity (60%) and temperature (25–28 °C), with a 12/12 h light/dark cycle. After acclimatization for 1 week, a total of 18 rats were randomly assigned into 3 groups with 6 rats in each group. All efforts were made to minimize the number of animals used and their suffering. All of the experimental procedures followed the Guide for the Care and Use of Laboratory Animals: Eighth Edition (ISBN-10: 0-309-15396-4).

### 4.2. ASD and CSD Models

MMPM is an established and commonly used animal model for studying REM SD. In the past, 72 h of continuous ASD and 21 days of non-continuous CSD were the classic models for sleep research. Rats were put into MMPM for 72 h to establish the ASD model and for 18 h/day for 21 days to establish CSD. The control and SD group rats were given 1 mL/day of double-distilled water by gavage, and the SD + TAK group rats were given 50 mg/kg/day TAK by gavage. The gavage was started 7 days before modeling to form pre-protection and maintain gavage during the modeling period.

### 4.3. Serum Test

The level of corticosterone (MM-0556R1, Jiangsu Enzyme Exemption Industry Co., Ltd., Yancheng, China), norepinephrine (MM-0559R1, Jiangsu Enzyme Exemption Industry Co., Ltd., Yancheng, China), glucose (B0668, Beckman Coulter Life Sciences, Suzhou, China), insulin (MM-0587R2, Jiangsu Enzyme Exemption Industry Co., Ltd., Yancheng, China), IL1β (MM-0922R1, Jiangsu Enzyme Exemption Industry Co., Ltd., Yancheng, China), IL10 (MM-0195R1, Jiangsu Enzyme Exemption Industry Co., Ltd., Yancheng, China), and TNF-α (MM-0180R1, Jiangsu Enzyme Exemption Industry Co., Ltd., Yancheng, China) were determined using the comprehensive instructions of Elisa Kits according to the manufacturer’s manual.

### 4.4. Liver Glycogen and Cholesterol Test

Liver glycogen (Nanjing Jiancheng Bioengineering institute, Nanjing, China, A043-1-1) and liver total cholesterol (Nanjing Jiancheng Bioengineering institute, Nanjing, China, A111-1-1) were measured according to the manufacturer’s manual.

### 4.5. Extraction of RNA and Quantitative Real-Time Quantitative PCR (qRT-PCR)

Total RNA from tissues and cells was isolated using TRIzol reagents (AG21102, Accurate Biology (HUNAN) Co., Ltd., Changsha, China), and reverse-transcribed into cDNA with Evo M-MLV RT Master Mix (AG11706, Accurate Biology (HUNAN) Co., Ltd., Changsha, China). The cDNA was then performed in quantitative real-time PCR in the BIO-RAD CFX96 qPCR Systems according to the manufacturer’s protocol. Actin was used as the housekeeping gene. The results were calculated by 2^−ΔΔCt^. Primer sequences are listed in Table 1.

### 4.6. Protein Extraction and Western Blot

Cells and tissues were thoroughly sonicated in immunoprecipitation buffer (Beyond, p0013, Midvale, UT, USA) with PMSF. After centrifuging at 15,000× *g* for 15 min at 4 °C, supernatants were collected, and protein concentration was measured with a BCA protein assay kit (Thermo Scientific, Waltham, MA, USA, no. 23229). The 10 μg protein samples were separated by 10% sodium dodecyl sulfate-polyacrylamide gel electrophoresis, transferred to pure nitrocellulose membranes (PerkinElmer Life Science, Boston, MA, USA), followed by standard immunoblotting procedures and analysis. The blots were developed with autoradiography films (Clinx Science Instruments, Shanghai, China). The band’s densitometry was analyzed through Clinx chemical analysis software.

The following antibodies were used: Anti-PI3K (110kD, Cell Signaling Technology, Boston, MA, USA, #4249), Anti-PI3K (85kD, Cell Signaling Technology, Boston, MA, USA, #4292), Anti-AKT (Cell Signaling Technology, Boston, MA, USA, #9272), Anti-Phospho-AKT (Ser473, Cell Signaling Technology, Boston, MA, USA, #9271), Anti-Phospho-GSK3 (Abcam, Cambridge, UK, #ab69476), Anti-GSK3 (Abcam, Cambridge, UK, #ab185141), Anti-AMPK (Cell Signaling Technology, Boston, MA, USA, #2531), Anti-Phospho-AMPK (Cell Signaling Technology, Boston, MA, USA, #2532), Anti-SREBP1 (Santa Cruz, Dallas, TX, USA, sc-365513), Anti-FASN (Cell Signaling Technology, Boston, MA, USA, #3180), Anti-ACC (Cell Signaling Technology, Boston, MA, USA, #3676), Anti-Phospho-ACC (Cell Signaling Technology, Boston, MA, USA, #2661), Anti-PPARα (Proteintech, Wuhan, China, #66826-1-lg), Anti-CPT1α (Proteintech, Wuhan, China, #15184-1-AP), Anti-NFκB p65 (Cell Signaling Technology, Boston, MA, USA, #8242), Anti-Phospho-NFκB p65 (Ser536, Cell Signaling Technology, Boston, MA, USA, #3033), Anti-β-ACTIN (Cell Signaling Technology, Boston, MA, USA, #4970), Anti-GAPDH (Cell Signaling Technology, Boston, MA, USA, #2118).

### 4.7. Histology and Immunostaining

Liver tissues were prepared for histology followed by the previous studies [34]. Hematoxylin and Eosin (H&E), Masson, Sirius Red, Oil Red, and Periodic Acid-Schiff (PAS) staining sections were processed by Wuhan Servicebio Technology Co., Ltd. (Wuhan, China). For fluorescence staining, cryostat sections were incubated with anti-CD68, anti-CD86, or anti-CD206 antibodies (Servicebio, Wuhan, China), followed by incubation with Alexa Fluor 488-conjugated or Alexa Fluor Cy3-conjugated secondary antibodies (Servicebio, Wuhan, China). Sections were evaluated under the bright-field or fluorescent microscope (Leica TCS SP8 STED 3X, Wetzlar, Germany).

### 4.8. Statistical Analysis

All data were presented as mean ± SEM. At first, the Kolmogorov–Smirnov test was used to verify if all data were normally distributed. One-way ANOVA was used to make group comparisons using GraphPad Prism 10. *p* values < 0.05 were considered statistically significant.

## 5. Conclusions

This study highlights the distinct patterns of liver injury induced by ASD and CSD and demonstrates the protective effects of TAK, a chalcone analog, against SD-induced hepatic damage. We observed that both ASD and CSD lead to weight loss, disruptions in rhythm disturbances, and elevated levels of norepinephrine and corticosterone, along with the expansion and fibrosis of the portal area. Moreover, glycogen synthesis disorder, lipid accumulation, and inflammation co-occur only in ASD. TAK effectively reversed the ASD-induced liver damage. This work provides valuable insight into liver pathology under SD and suggests potential therapeutic strategies for the prevention and treatment of liver damage associated with SD.

## Figures and Tables

**Table 1:** Gene list.

Gene Name	Primer Sequence	
*Per1*	Forward	TACCAGCCATTCCGCCTAAC
*Per1*	Reverse	CCGGGGAGCTTCATAACCAG
*Per2*	Forward	CGAAGCGCCTCATTCCAGAG
*Per2*	Reverse	TGCTCATGTCCACGTCTTCC
*Gys2*	Forward	GTGTGACTACGAACCTCTC
*Gys2*	Reverse	CCTCCTCTTCCTCATCATATC
*Slc2a1*	Forward	TGGCCAAGGACACACGAATACTGA
*Slc2a1*	Reverse	TGGAAGAGACAGGAATGGGCGAAT
*Hk2*	Forward	CGAGAGCATCCTCCTCAAGTG
*Hk2*	Reverse	AGCCACAGGTCATCATAGTTCC
*Pkm2*	Forward	TGGGAGAGAAGGGAAAGAACATC
*Pkm2*	Reverse	GCACCGTCCAATCATCATCTTC
*Ldha*	Forward	ATGAGTTGGACTGTGCCTGTTGTG
*Ldha*	Reverse	GTGAAGAGCCAGGTGCCGTTG
*Cs*	Forward	CATACTGAGCAATCTGATACC
*Cs*	Reverse	GAGCCAAGAGACCTGTTC
*Idh3*	Forward	ACCAATACAGAGCAACAGAT
*Idh3*	Reverse	GCACATCACCATCATAGTTC
*Ogdh*	Forward	ATGATGGGCAGCAAGATG
*Ogdh*	Reverse	TTCCTCCTGAGACATAGGTT
*Nos2*	Forward	GCATCCCAAGTACGAGTGGT
*Nos2*	Reverse	GAAGGCGTAGCTGAACAAGG
*Arg1*	Forward	GGACATCGTGTACATCGGCT
*Arg1*	Reverse	GTAGCCGGGGTGAATACTGG

## Data Availability

The authors confirm that the data supporting the findings of this study are available within the article.

## Supplementary Materials

The following supporting information can be downloaded at: https://www.mdpi.com/article/10.3390/ijms26083485/s1.

## Abbreviations

The following abbreviations are used in this manuscript:

ACC	Acetyl CoA Carboxylase
ASD	Acute Sleep Deprivation
CPT1A	Carnitine Palmitoyl Transferase 1A
CSD	Chronic Sleep Deprivation
CS	Citrate Synthase
FASN	Fatty Acid Synthase
GLUT1	Glucose Transporter 1
GYS2	Glycogen Synthase 2
GSK3	Glycogen Synthase Kinase-3
H&E	Hematoxylin and Eosin
HSCs	Hepatic Stellate Cells
HK2	Hexokinase 2
IDH	Isocitrate Dehydrogenase
KCs	Kupffer Cells
LDHA	Lactate Dehydrogenase A
MMPM	Modified Multi-Platform Method
NREM	Non-Rapid Eye Movement
Nrf2	Nuclear Factor E2-Related Factor 2
PAS staining	Periodic Acid-Schiff Staining
PI3K	Phosphoinositide 3-kinase
AKT	Protein Kinase B
PKM2	Pyruvate Kinase M2
qRT-PCR	Quantitative real-time polymerase chain reaction
REM	Rapid Eye Movement
SREBP	S1P Mediated Cholesterol Regulatory Element Binding Protein
UDPG	Uridine Diphosphate Glucose
α-SMA	α-Smooth Muscle Actin
α-KGDH	α-Ketoglutarate Dehydrogenase
