# Supplementary material for: Distinct Hepatic Metabolic Reprogramming in Acute and Chronic Sleep Deprivation and the Protective Effects of the Chalcone Analogue TAK
Source: Int J Mol Sci. 2025 Apr 8;26(8):3485. doi: 10.3390/ijms26083485 (PMC12027424; doi:10.3390/ijms26083485)
Supplement: Supplementary file 1 [file ijms-26-03485-s001.zip › ijms-3500167-supplementary.pdf]

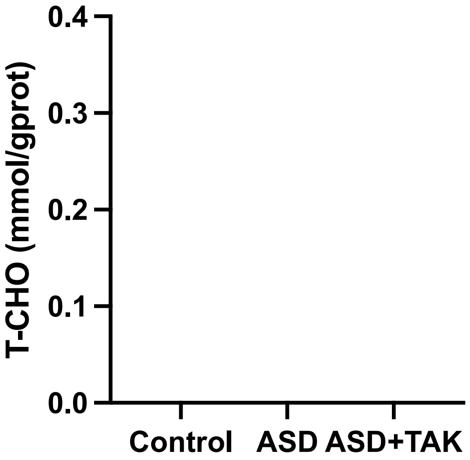

Supplement Figure 1. The cholesterol content of liver tissue (n = 6). Data are presented as mean ± SEM. Statistical analysis was performed using one-way ANOVA with Dunnett's test, performed with GraphPad Prism 10. Significance levels are indicated as follows: * $p < 0.05$, ** $p < 0.01$, *** $p < 0.001$.
